# Googling for Suicide–Content and Quality Analysis of Suicide-Related Websites: Thematic Analysis

**DOI:** 10.2196/29146

**Published:** 2021-11-11

**Authors:** Wen Chen, Andrea Boggero, Giovanni Del Puente, Martina Olcese, Davide Prestia, Haitham Jahrami, Nasr Chalghaf, Noomen Guelmami, Fairouz Azaiez, Nicola Luigi Bragazzi

**Affiliations:** 1 Department of Psychiatry Xiamen Xianyue Hospital Xiamen China; 2 Department of Educational Science University of Genoa Genoa Italy; 3 Department of Neuroscience, Rehabilitation, Ophthalmology, Genetics and Maternal/Child Sciences University of Genoa Genoa Italy; 4 Department of Psychiatry Istituto di ricovero e cura a carattere scientifico Ospedale Policlinico San Martino Genoa Italy; 5 College of Medicine and Medical Sciences Arabian Gulf University Manama Bahrain; 6 Ministry of Health Manama Bahrain; 7 Higher Institute of Sport and Physical Education of Sfax University of Sfax Sfax Tunisia; 8 Higher Institute of Sport and Physical Education of Kef University of Jendouba Jendouba Tunisia; 9 Laboratory for Industrial and Applied Mathematics Department of Mathematics and Statistics York University Toronto, ON Canada

**Keywords:** suicide, internet, world wide web, content analysis, HONcode, mental health, webpage, health information, eHealth

## Abstract

**Background:**

Suicide represents a public health concern, imposing a dramatic burden. Prosuicide websites are “virtual pathways” facilitating a rise in suicidal behaviors, especially among socially isolated, susceptible individuals.

**Objective:**

The aim of this study is to characterize suicide-related webpages in the Italian language.

**Methods:**

The first 5 most commonly used search engines in Italy (ie, Bing, Virgilio, Yahoo, Google, and Libero) were mined using the term “suicidio” (Italian for suicide). For each search, the first 100 webpages were considered. Websites resulting from each search were collected and duplicates deleted so that unique webpages could be analyzed and rated with the HONcode instrument

**Results:**

A total of 65 webpages were included: 12.5% (8/64) were antisuicide and 6.3% (4/64) explicitly prosuicide. The majority of the included websites had a mixed or neutral attitude toward suicide (52/64, 81.2%) and had informative content and purpose (39/64, 60.9%). Most webpages targeted adolescents as an age group (38/64, 59.4%), contained a reference to other psychiatric disorders or comorbidities (42/64, 65.6%), included medical/professional supervision or guidance (45/64, 70.3%), lacked figures or pictures related to suicide (41/64, 64.1%), and did not contain any access restraint (62/64, 96.9%). The major shortcoming to this study is the small sample size of webpages analyzed and the search limited to the keyword “suicide.”

**Conclusions:**

Specialized mental health professionals should try to improve their presence online by providing high-quality material.

## Introduction

Suicide represents a public health concern, imposing a dramatic burden both in epidemiological and societal terms [1]. According to the latest available 2021 report of the World Health Organization (WHO) in 2019, Italy had a crude rate of 6.7 (95% CI 6.0-8.5) suicides per 100,000 people, with a male:female ratio of 2.9 [2].

Within the current eHealth era, in which the new information and communication technologies are being increasingly exploited to obtain health-related information [3], the internet can be characterized as having a Yin-Yang, paradoxical nature [4]. On the one hand, there exists a statistically significant correlation between a pathological use of the web and suicidality (either in terms of suicidal ideation or nonsuicidal self-injury). Prosuicide websites and online suicide pacts are “virtual pathways” that facilitate the emergence of suicidal behaviors, especially among socially isolated and susceptible individuals. On the other hand, the internet can serve as an effective tool for suicide monitoring and tracking [3,5] and can play a major role in suicide prevention [6,7], particularly for those vulnerable individuals who would be otherwise difficult to reach with conventional approaches [8,9].

Understanding the determinants of suicide-related web searches is, therefore, of crucial importance [10]. According to Wong and collaborators [11], the categories of the most visited suicide-related webpages can be classified into entertainment (30.13%), scientific information (18.31%), and community resources (14.53%). Among the 1314 accessed webpages in their study, prosuicide websites represented only a small fraction (0.15%). The most used search terms were “committing suicide with a gas oven,” “hairless goat,” “pictures of murder by strangulation,” and “photo of a severe burn.”

Suicide-related digital behavior has been assessed in different countries and different languages [12-15]. However, to the best of our knowledge, there is a dearth of information concerning suicide-related webpages in the Italian language. As such, the aim of this study is to address this gap in knowledge.

## Methods

### Suicide-Related Website Selection and Inclusion

The first 5 most commonly used search engines in Italy (namely, Bing, Virgilio, Yahoo, Google, and Libero) were mined with the search term “suicidio” (Italian for suicide). For each search, the first 100 webpages were considered. Websites resulting from each search were collected and duplicates deleted so that webpages could be analyzed and rated by 2 authors independently (MO and DP).

Quality assessment of the included webpages was performed with the HONcode instrument, which comprises the following sections: authoritative (if the qualifications of the authors are mentioned), complementarity (if the site aims at supporting but not replacing or substituting the physician-patient relationship), privacy (if visitors' data are protected and respected), attribution (if sources of published information in the medical and health-related pages are explicitly mentioned and referred to), justifiability (if claims related to potential benefits or performance are evidence based), transparency (if the presentation of the online material is accessible, with accurate email contacts), financial disclosure (if eventual funding sources are identified and mentioned), and advertising policy (if advertising and editorial materials are separate and identifiable) [16].

The agreement between the 2 authors was assessed with the Cohen κ statistic.

Furthermore, the 2 authors filled in an ad hoc predesigned Excel spreadsheet (Microsoft Corp) containing questions concerning the attitude of the webpage toward suicide (prosuicide, antisuicide, mixed or neutral), content and purpose of the webpage (supporting or emotional, informative, preventive), supervision or guidance (if the webpage was user-generated or handled by a professional figure, such as a psychiatrist or a psychologist), reference to age groups (adolescents or older people) targeted or referred to by the webpage (this simple dichotomic stratification was applied based on the availability of information provided and considering that the majority of internet users consist of adolescents), reference to other psychiatric disorders or comorbidities, the presence of figures or pictures related to suicide, and the presence of access restraint.

Moreover, for each included webpage, the number of comments’ likes, and shares, if any, was recorded.

### Statistical Analysis

Before commencing any statistical handling and processing of the data, figures were visually inspected for potential outliers. The normality of data distribution was checked using the D’Agostino-Pearson omnibus test. Univariate analysis (chi-squared test and the chi-squared test for trend, Fisher’s exact test, *t* test, and analysis of variance or their nonparametric versions in case of violation of normal distribution) was performed for the variables under study. Multivariate linear regression analysis was performed to shed light on the determinants of the HONcode score.

All statistical analyses were performed with the commercial SPSS for Windows, version 24.0 (IBM Corp). Figures with *P* values less than .05 were considered statistically significant.

## Results

Sixty-four unique websites were retrieved and analyzed. Concerning the attitude toward suicide, 4 webpages were in favor of suicide (6.3%), whereas 8 sites were against suicide (12.5%). The remaining webpages (52/64, 81.2%) were neutral toward suicide. The overrepresentation of neutral pages in tone was statistically significant (*χ*^2^_2_=30.55; contingency coefficient=0.44; *P*<.001; *χ*^2^_1_ for trend=28.40; *P*<.001). In terms of content and purpose, 6 webpages were supporting or emotional (9.4%), 39 informative (60.9%), and 19 preventive (29.7%), with both emotional and preventive pages being underrepresented (*χ*^2^_2_=13.83, contingency coefficient=0.31, *P*=.001; *χ*^2^_1_ for trend=13.67, *P*<.001). Concerning the supervision or guidance of the websites, 45 (70.3% of the entire sample) were handled by a professional figure (a medical doctor or a psychologist, or an allied health professional). Consequently, the medical supervision or guidance of the webpages was predominant (*P*=.03). Thirty-eight webpages were targeted to or contained references to adolescents as an age group particularly vulnerable to suicide (59.4%; 26/64 websites referred to older people, 40.6%; *P*=.37). Forty-two (65.6%) webpages made references to other psychiatric disorders or comorbidities, such as depression (65.6%) or drug use (26.6%), while twenty-two (34.4%) websites did not. Webpages mentioning comorbidities were slightly overrepresented (*P*=.07). Twenty-three webpages (35.9%) versus forty-one (64.1%) contained figures or pictures related to suicide (not statistically significant, *P*=.15). Sixty-two webpages (96.9% of the sample) had no access restraint (*P*<.001).

The average HONcode score was 5.81 (SD 2.70, median 7), whereas the average overall number of likes, comments, and shares was 14,261.56 (SD 60,065.23, median 15.00). The correlation between HONcode and the overall number of likes, comments, and shares was 0.16 (95% CI –0.33 to 0.58; *P*=.53). Further details are reported in Table 1.

Webpages were generally compliant with the HONcode principles (range 68.8%-100.0%; authoritative principle: 44/64; principle of transparency: 64/64) except for the principle of justifiability (35/64, 54.7%, *P*=.72) and financial disclosure (40/64, 62.5%, *P*=.21), as shown in Table 2.

At the univariate analysis (Table 3), the HONcode score was higher in mixed or neutral webpages (*P*<.001), in websites with a preventive purpose (*P*=.02), with professional or medical supervision or guidance (*P*<.001), with references to other psychiatric disorders or comorbidities (*P*=.003), and without access restraint (*P*=.03). No statistically significant differences could be detected in terms of references to age groups (adolescents versus older people, *P*=0.81) or the presence of figures or pictures (*P*=.41).

Furthermore, webpages with medical supervision or guidance had more an informative and preventive content and purpose than did supporting or emotional ones (*χ*^2^_2_=9.19, *P*=.01; *χ*^2^_1_ for trend=3.19, *P*=.07). Those webpages with professional supervision or guidance also had a more positive or mixed/neutral attitude toward suicide, and this was statistically significant (*P*=.002). In contrast, no differences were found in terms of references to other psychiatric disorders or comorbidities, references to age groups, access restraint, and the presence of figures or pictures related to suicide.

In the multivariate linear regression analysis (Table 4), the predictors of the HONcode score were professional/medical or supervision/guidance of the webpages (regression coefficient=3.94; *P*<.001) and references to psychiatric disorders as comorbidities (regression coefficient=1.46; *P*=.003).

Meanwhile, no differences were found in terms of likes, comments, or shares either in the univariate or the multivariate analyses.

**Table 1:** Characteristics of the included websites (N=64).

Parameter			Value
**Attitude toward suicide, n (%)**			
	Prosuicide	4 (6.3)	
	Antisuicide	8 (12.5)	
	Mixed/neutral	52 (81.2)	
**Content and purpose, n (%)**			
	Supporting/emotional	6 (9.4)	
	Informative	39 (60.9)	
	Preventive	19 (29.7)	
**Supervision/guidance, n (%)**			
	Religious figure	4 (6.3)	
	Psychiatrist	11 (17.2)	
	Other kinds of physicians	3 (4.7)	
	Psychologist	25 (39.1)	
	Pharmacist	1 (1.6)	
	Other allied health professionals	5 (7.8)	
	Lawyer	1 (1.6)	
	User-generated webpage	11 (17.2)	
	Volunteer organization/charity	3 (4.7)	
**References to age groups, n (%)**			
	Adolescents	38 (59.4)	
	Older people	26 (40.6)	
**Reference to other psychiatric disorders, n (%)**			
	Depression	42 (65.6)	
	Anxiety	11 (17.2)	
	Panic disorder	2 (3.1)	
	Bipolar syndrome	9 (14.1)	
	Personality disorder	8 (12.5)	
	Posttraumatic stress disorder	3 (4.7)	
	Paranoia	2 (3.1)	
	Schizophrenia	12 (18.8)	
	Drug use	17 (26.6)	
	Alcoholism	6 (9.4)	
	Eating and weight disorders	1 (1.6)	
	Sleep disorders	1 (1.6)	
	Reference	42 (65.6)	
	No reference	22 (34.4)	
**Figures/pictures, n (%)**			
	Yes	23 (35.9)	
	No	41 (64.1)	
**Access restraint, n (%)**			
	Yes	2 (3.1)	
	No	62 (96.9)	
HONcode score, mean (SD)			5.81 (2.7)
Comments, n			585
Shares, n			98
Likes, n			256,025

**Table 2:** Assessment of the suicide-related websites according to the HONcode principles (N=64).

HONcode principle	Yes	No	*P* value
1. Authoritative	44 (68.8)	20 (31.2)	.047
2. Complementarily	48 (75.0)	16 (25.0)	.006
3. Privacy	48 (75.0)	16 (25.0)	.006
4. Attribution	45 (70.3)	19 (29.7)	.03
5. Justifiability	35 (54.7)	29 (45.3)	.72
6. Transparency	64 (100.0)	0 (0.0)	<.001
7. Financial disclosure	40 (62.5)	24 (37.5)	.21
8. Advertising policy	50 (78.1)	14 (21.9)	.002

**Table 3:** Univariate analysis of the studied webpages according to the HONcode score.

Parameter		HONcode score, mean (SD)		*P* value
**Attitude toward suicide**				<.001
	Prosuicide	0.75 (0.50)		
	Antisuicide	3.50 (2.93)		
	Mixed/neutral	6.55 (2.07)		
**Content and purpose**				.02
	Supporting/emotional	2.17 (2.93)		
	Informative	6.08 (2.45)		
	Preventive	6.42 (2.22)		
**Supervision/guidance**				<.001
	User-generated webpage	2.63 (2.27)		
	Professional figures	7.16 (1.38)		
**References to age groups**				.81
	Adolescents	6.05 (2.36)		
	Older people	5.46 (3.09)		
**Reference to other psychiatric disorders**				.003
	No reference	4.28 (3.16)		
	Reference	6.41 (2.22)		
**Figures/pictures**				.41
	Yes	6.09 (2.83)		
	No	5.66 (2.60)		
**Access restraint**				.03
	Yes	100 (0.00)		
	No	5.97 (2.57)		

**Table 4:** Multivariate linear regression analysis of the studied webpages according to the HONcode score.

Independent variables	Value				
	Coefficient	SE	r_partial_	*t*	*P*
Constant	1.11	N/A^a^	N/A	N/A	N/A
Content and purpose	0.27	0.20	0.17	1.30	.20
Reference to age groups	0.18	0.44	0.06	0.42	.78
Figures/pictures	0.40	0.45	0.12	0.89	.38
Supervision/guidance	3.94	0.47	0.75	8.30	<.001
Access restraint	–1.99	1.42	–0.19	–1.40	.17
References to other psychiatric disorders	1.46	0.47	0.39	3.12	.003
Attitude toward suicide	0.52	0.57	0.12	0.91	.37

^a^N/A: not applicable.

## Discussion

### Main Findings and Comparison With the Literature

To the best of our knowledge, this is the first study to assess the quality and content of suicide-related webpages in the Italian language.

We found that the percentage of explicitly prosuicide websites was greater than that reported by Wong et al [11] but less than that reported by Chen et al (16.3%), who analyzed 375 linked webpages in Chinese [12]. According to the authors, 41.3% of the included webpages were antisuicide, and the majority of the prosuicide sites were user-generated (96.7%). Interestingly, searches using the search term “ways to kill yourself” (31.7%) and “painless suicide” (28.3%) generated much larger numbers of potentially harmful webpages than did the keyword “suicide” (4.3%).

A much larger portion of prosuicide websites (42%) was found by Sakarya et al [17], who analyzed 100 webpages. Thirteen percent of the studied sample was found to have content or material that may be considered protective against suicidal behavior, a result in line with our findings. However the authors found that “protective” websites did not have any kind of medical supervision or guidance from specialized mental health professionals. In contrast to this, we managed to find a statistically significant association between these 2 variables.

Til and Niederkrotenthaler [18] performed a cross-cultural study by comparing the suicide-related web searches in Austria and the USA. They found that in both countries, protective outweighed harmful website characteristics by approximately 2:1, a finding in line with our result. Authors were able to confirm the observation of Wong et al: websites retrieved with method-related search terms (eg, how to hang yourself) contained more harmful information (USA: *P*<.001; Austria: *P*<.05) and fewer protective characteristics (USA: *P*<.001; Austria: *P*<.001) compared to the term “suicide” [11]. Conversely, help-related search terms (eg, suicidal thoughts) yielded more webpages with positive and protective characteristics (USA: *P*=.07; Austria: *P*<.01). Interestingly, authors found that websites retrieved with the US search engines generally had more protective characteristics (*P*<.001) than did those retrieved with Austrian search engines. Moreover, resources with dangerous or harmful characteristics were better ranked than those with positive or protective characteristics (USA: *P*<.01; Austria: *P*<.05).

In another study, Thornton and colleagues [13] found that among Google searches retrieved related to suicide, a high portion of them were irrelevant webpages (n=136, approximately 26% of the entire sample). Of the 329 relevant websites, the majority were suicide preventive (about 68%); however, a considerable proportion of sites expressed mixed/ambiguous (22%) or neutral (8%) attitudes toward suicide, and 1% were explicitly prosuicide, figures which were slightly different from ours.

These inconsistent literature findings could be explained by the cultural differences across countries in which suicide-related web searches are performed, as shown by the research of Till and Niederkrotenthaler [15], as well as policies concerning the web. Overall, the combination of societal data and online behavior monitoring provide the best indication of risks [19].

The presence of antisuicide online material is of crucial importance for 2 major reasons: (1) according to a recent review of the literature [6], individuals at risk for suicide are probably more prone to look for suicide-related information online, and searching online information related to suicide is a proxy of suicidal ideation; and (2) according to a randomized controlled trial, education professional suicide prevention websites appear to increase and improve suicide prevention–related knowledge, especially among vulnerable and socially isolated individuals [18]. Therefore, exposure to high-quality curated websites may be associated with a reduction in suicidal thoughts and actions. Recent research has shown that the proactive suicide prevention online model is robust for identifying people that are at risk of suicide [20].

### Strengths and Limitations of the Study

Despite some strengths, including being the first study to systematically assess the quality of suicide-related webpages in the Italian language, our investigation suffers from some limitations that should be properly acknowledged. The major shortcoming is the small sample size of webpages analyzed and the search limited to the keyword “suicide.” Future research should also explore other words indirectly related to suicide as well as other terms related to the ways suicide can be committed (ie, method-related search terms) and explore whether the semantic differential has an impact on suicide-related webpages in terms of content and quality. Moreover, caution should be used when generalizing the present findings to other countries, as our results could be affected by country-specific cultural issues and may not reflect the nature of other settings. Further, although we assessed the reference to other psychiatric disorders or comorbidities in the webpages included in our study, we were not able to establish a link between mental illness and suicide in terms of browsing websites. This warrants dedicated, ad hoc studies specifically exploring this topic. Another aspect that should be investigated concerns the size or burden of the psychological effect imposed by browsing suicide-related websites.

### Conclusions

Sixty-four webpages were included in our analysis: 12.5% (8/64) were antisuicide and 6.3% (4/64) explicitly prosuicide. The majority of the included websites had a mixed or neutral attitude toward suicide (52/64, 81.2%) and had an informative content and purpose (39/64, 60.9%). Most webpages targeted adolescents as an age group (38/64, 59.4%), contained a reference to other psychiatric disorders or comorbidities (42/64, 65.6%), had medical or professional supervision or guidance (45/64, 70.3%), lacked figures or pictures related to suicide (41/64, 64.1%), and did not use any access restraint (62/64, 96.9%).

Specialized mental health professionals should take into account these findings, make an effort to improve their presence online, and provide high-quality material. However, given the aforementioned limitations, further research in the field is warranted.

## Abbreviations

WHO: World Health Organization
